# Supplementary material for: Human noise blindness drives suboptimal cognitive inference
Source: Nat Commun. 2019 Apr 12;10:1719. doi: 10.1038/s41467-019-09330-7 (PMC6461696; doi:10.1038/s41467-019-09330-7)
Supplement: Supplementary file 1 — Supplementary Information [file 41467_2019_9330_MOESM1_ESM.pdf]

Supplementary Information

Human noise blindness drives suboptimal cognitive inference

Herce Castañón *et al*.

Supplementary Methods

In all experiments, participants had to judge the average orientation of an array of gratings as clockwise (CW) or counter-clockwise (CCW) relative to horizontal. We first describe trial events, trial timings and stimulus construction for Experiment 1 and then explain the additional steps taken for Experiments 2-6.

In Experiment 1, a fixation dot first appeared at the centre of the screen for 300 ms to announce the start of a trial. The fixation dot was replaced by a cue which appeared 700 ms before the stimulus array and which remained on the screen until a response was registered. The cue determined the prior probability of each stimulus category ("L": prior probability of CCW is 75%; "N": CCW and CW equally likely; "R": prior probability of CW is 75%). The stimulus array was shown for 150 ms and was followed by a response window lasting up to 3000 ms. Participants responded by pressing the left (CCW) or right (CW) arrow-key on a QWERTY keyboard using their right hand. Categorical feedback about choice accuracy ("CORRECT" or "WRONG") appeared once a response had been registered and remained on the screen for 500 ms, before the onset of the next trial. If no response was registered within the response window, the word "LATE" appeared at the centre of the screen for 3000 ms, and the next trial was automatically started.

The stimulus consisted of eight gratings displayed within a circular array. We manipulated two features of the stimulus array in a factorial manner: the contrast of the gratings and the variability of the gratings' orientations.

The centre of each grating was located at a distance of ~4.3 degrees of visual angle (400 pixels) from the centre of the screen. Each grating was a Gabor patch constructed using the following parameter values: diameter of ~1.07 degrees of visual angle (100 pixels); spatial frequency of ~5 cycles per degree of visual angle (0.05 cycles per pixel); random phase. All gratings had the same root mean square contrast (rmc, henceforth contrast). The contrast of a trial was either 0 (no signal), .15 (low contrast) or .60 (high contrast). The latter two contrast levels may not affect orientation discrimination on their own[1]. However, we added low-level random noise to the gratings [2]. For each grating, we convolved a unique patch of white noise with a two-dimensional zero-mean Gaussian with a standard deviation of ~0.21 degrees of visual angle (20 pixels). The amplitude of the noise was 10% of the maximum possible. We then added the noise to the grating. Finally, we convolved the grating with a 2-dimensional Gaussian envelope peaking at the centre of the grating and decaying with a standard deviation of ~0.21 degrees of visual angle (20 pixels).

The average orientation of gratings on a trial (henceforth trial mean) was randomly drawn from a Gaussian distribution with a mean of -/+ 3º and a standard deviation of 8º. The variability in the orientations of gratings on a trial (henceforth variability) was randomly drawn from a Gaussian distribution with a mean 0º and a standard deviation of either 0º (zero variability), 4º (medium variability) or 10º (high variability). To ensure the trial mean remained unchanged after the variability manipulation, we subtracted the mean deviation from 0 from the gratings' orientations. Together, these steps allowed us to independently manipulate trial mean, contrast and variability. We emphasise that feedback was determined by the average orientation of the gratings presented and not by the distribution from which they were drawn.

The experiment consisted of 1296 trials, distributed into 36 blocks of 36 trials each. On half of the blocks, the prior cue was "N" (neutral trials). On the other half of blocks, the cue varied randomly between "L" or "R" (biased trials). Block order was randomised across an experiment and across participants.

In Experiment 2, we introduced an explicit measure of confidence. Participants indicated their choice by pressing "Z" (CCW) or "X" (CW) using their left hand. After having made a choice, participants were asked to indicate the probability that the choice is correct. Participants indicated their estimate by sliding a marker along a vertical scale (50% to 100% in increments of 1%) using a standard computer mouse with their right hand. The probability associated with the marker's current position was updated in real-time and shown at the centre of the screen. Participants confirmed their response by clicking the left button of the mouse. There was no time limit for the confidence judgment. Feedback about choice accuracy appeared 300 ms after a response had been confirmed. The experiment consisted of 1296 trials, distributed into 36 blocks of 36 trials each. On half of the blocks, the prior cue was "N" (neutral trials). On the other half of blocks, the cue varied randomly between "L" or "R" (biased trials). Block order was randomised across an experiment and across participants.

In Experiment 3, we introduced an implicit measure of confidence. On half of the blocks, participants could choose to opt out of making a choice and receive the same reward as a correct choice with a 75% probability. To remind participants about the choice options on a trial, the words "LEFT" (CCW) and "RIGHT" (CW) appeared to the left and the right of the fixation cross after the stimulus disappeared and, when the opt-out option was available, the words "OPT OUT" appeared below the fixation cross. The opt-out option was selected by pressing the downwards arrow key. For feedback, "SUCCESS" was shown after a correct choice and a rewarded opt-out response, whereas "FAILURE" was shown after an incorrect choice and an unrewarded opt-out response. The experiment consisted of 1296 trials, distributed into 36 blocks of 36 trials each. On half of the blocks, the opt-out option was not available. On the other half of the blocks, the opt-out option was available. Block order was randomised across an experiment and across participants. There was no prior cue.

In Experiment 4, we asked participants to categorise either the contrast (rmc = {.15, .60}) or the variability (std = {0º, 10º}) of the stimulus array. In particular, after having made a choice (by pressing the "X" and "Z" buttons using their left hand, with the chosen category highlighted in bold), participants were then required to judge whether the contrast of the stimulus array was high or low or whether the variability of the stimulus array was high or low. The relevant stimulus dimension for the second judgment (indicating by displaying "CONTRAST" or "VARIABILITY at the centre of the screen), was determined randomly and was only revealed after an orientation discrimination had been made. Participants made the second judgment by pressing the left (low) or the right (high) arrow key (the options "LOW" and "HIGH" appeared equidistantly to the left and the right of the fixation point). Once participants had made their response, they received feedback about the accuracy of each judgment, indicated by changing the colours of the selected options to red (incorrect) or green (correct). The experiment consisted of 1200 trials, distributed into 32 blocks of 40 trials each. On half of the blocks, the prior cue was "N" (neutral trials). On the other half of blocks, the cue varied randomly between "L" or "R" (biased trials). Block order was randomised across an experiment and across participants.

In Experiment 5, we fixed either contrast or variability across blocks of trials. Specifically, within a block, one dimension was fixed, while the other dimension varied randomly. For instance, in one condition, contrast would be fixed at low while variability varied between high and low across the trials within the block. As in Experiment 4, there were only two levels of contrast and two levels of variability. There were thus four trial types in total. On half of the blocks for a trial type, the prior cue was "N" (neutral trials). On the other half of blocks for a trial type, the prior cue varied randomly between "L" or "R" (biased trials). The experiment consisted of 1200 trials,

distributed into 32 blocks of 40 trials each. There were eight blocks for each trial type. Block order was randomised across an experiment and across participants.

In Experiment 6, we varied the set-size of the stimulus array. In particular, the stimulus array was composed of either four or eight gratings. As in Experiments 4 and 5, there were only two levels of contrast and two levels of variability. For arrays with only four gratings, the location of the gratings was fixed within a block but randomised across blocks, by sampling a random set of four contiguous locations from the full array with eight gratings. The set-size was varied in a trial-by-trial manner. Half of the trials had a set-size of four gratings and the other half of trials had a set-size of eight gratings. We scaled the variance of the distribution of orientation variabilities for the four-item set size to ensure that the observed standard deviation of orientations within a set-size was equated for the four-item and the eight-item cases. Without this step, the observed variance of the four-item set-size would be systematically lower than for the eight-item set-size. The experiment consisted of 1200 trials, distributed into 32 blocks of 40 trials each. On half of the blocks, the prior cue was "N" (neutral trials). On the other half of blocks, the cue varied between randomly between "L" or "R" (biased trials). Block order was randomised across an experiment and across participants.

All participants were reimbursed for their participation and had the opportunity to earn an additional performance-based bonus. In all experiments except Experiment 2, participants received a flat rate of £10 and could earn an additional £1 for every 2% increase in choice accuracy relative to 60%. In Experiment 2, participants received a flat rate of £5 and could earn an additional bonus depending on the accuracy of their confidence judgments. We submitted participant' responses to a strictly proper scoring rule under which it was in participants' best interest to make as many correct decisions as possible and to estimate the probability that their choice is correct as accurately as possible[3]. The average bonus accrued was ~£12.

Participants received a 10-minute introduction to the task, including the stimulus, sources of choice difficulty, prior cue and prior probabilities, response contingencies, and the rule for calculating the performance-based bonus. Participants also completed a short practice session (two blocks) before starting the experiment proper.

Supplementary Note 1

Comparison of computational models

We considered a set of models which have the same *generative* model of how sensory evidence is generated but differ in their *internal* model of this process. In particular, they differ with respect to (i) an agent's ability to identify which condition a trial is drawn from and (ii) an agent's sensitivity to the different sources of noise in play. These differences give rise to different inferences (all trials) and thereby different responses (biased trials). In Supplementary Table 1, we provide model names (column 1), quantitative comparison against the best-fitting noise-blind model (column 2), and details about model assumptions (columns 3-9). Note that the number of unique pairs of category-conditioned PDFs depends on the experiment (the first number is for Exp1-3; the number in brackets is for Exp4-6). For completeness, we considered models which intuitively seem unlikely (e.g., the Full Mixer model which cannot discriminate experimental conditions).

Supplementary Figure 1

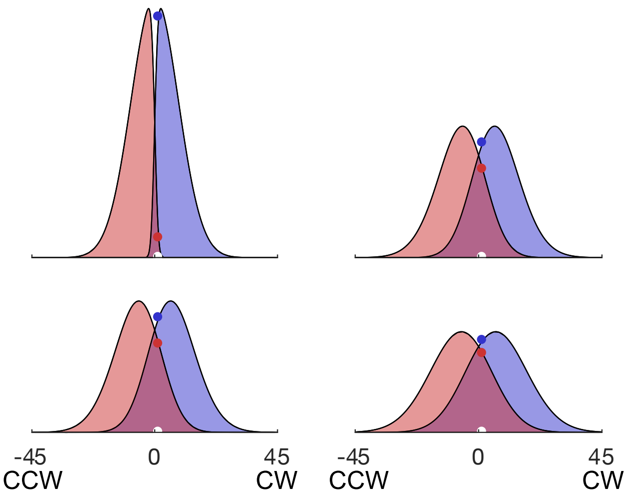

Category-conditioned probability density functions. Here we illustrate category-conditioned probability density functions for an omniscient agent. We assumed that an omniscient agent's internal model has, for each experimental condition, a unique pair of category-conditioned probability density functions (PDFs) over sensory evidence. Four pairs of PDFs are shown, one for each of the four conditions in Experiment 4-6. Note that the PDFs look more skewed in conditions with low noise (top-left) as they will more closely resemble the true distribution of average orientations (Fig. 4a). Top-left: high-contrast and low-variability trials (lowest noise). Top-right: high-contrast and high-variability trials (intermediate noise). Bottom-left: low-contrast and low-variability trials (intermediate noise). Bottom-right: low-contrast and high-variability trials (highest noise). The white dots on the x-axes denote an agent's current sensory evidence (same across panels). The blue and red dots indicate the probability density that the sensory evidence came from a CW or a CCW category, respectively.

Supplementary Table 1

Model Name	Average BIC difference with best	Number of unique pairs of PDFs	Is blind to Encoding Noise?	Knows the contrast condition of each trial?	Is blind to Integration Noise?	Knows the variability condition of each trial?	Operates with the average Encoding Noise?	Operates with the average Integration Noise?
Omniscient	-32.98	6 (4)	No	Yes	No	Yes	No	No
Noise Blind	0.00	2 (2)	No	Yes	**Yes**	**Irrelevant**	No	No
Variability Mixer	-20.41	2 (2)	No	Yes	No	**No**	No	No
Contrast Mixer	-60.39	3 (2)	No	**No**	No	Yes	No	No
Full Mixer	-58.57	1 (1)	No	**No**	No	**No**	No	No
Average Variability	-47.28	2 (2)	No	Yes	No	**No**	No	**Yes**
Average Contrast	-116.93	3 (2)	No	**No**	No	Yes	**Yes**	No
Full Average	-134.76	1 (1)	No	**No**	No	**No**	**Yes**	**Yes**
Contrast Blind	-9.79	3 (2)	**Yes**	**Irrelevant**	No	Yes	No	No

Model assumptions and model comparison. Model comparison was based on the difference in average BIC across participants (Exp1-3) relative to the noise-blind model.

Supplementary Note 2

Modelling inference using the full stimulus array

Here we describe an *ensemble* model which makes inferences using eight noisy samples, one sample describing the orientation of each grating, rather than one noisy sample describing the

average orientation of the full stimulus array. We first describe the generative model of how sensory evidence arises at the level of single items, and then describe the internal model used to make inferences based on sensory evidence.

On each trial, we first sampled the true average orientation of the stimulus array, x', from a Gaussian distribution with mean, $\mu = \pm 3$, and variance, $\sigma^2 = 8$: $x' \sim N(\mu, \sigma^2)$. The sign of x' defined the stimulus category. Hence, the distribution of x' given each category is:

$$\mathrm{p}(x'|\mathrm{CW}) = \left\{ \begin{matrix} \frac{1}{2} * \frac{1}{\sqrt{16\pi}} * (\mathrm{e}^{-\frac{(x'-\mu)^2}{16}} + \mathrm{e}^{-\frac{(x'+\mu)^2}{16}}) & x' \geq 0 \\ 0 & x' < 0 \end{matrix} \right\} \tag{1}$$

$$\mathrm{p}(x'|\mathrm{CCW}) = \left\{ \begin{matrix} \frac{1}{2} * \frac{1}{\sqrt{16\pi}} * (\mathrm{e}^{-\frac{(x'-\mu)^2}{16}} + \mathrm{e}^{-\frac{(x'+\mu)^2}{16}}) & x' < 0 \\ 0 & x' \geq 0 \end{matrix} \right\} \tag{2}$$

We then generated eight samples, $u_{\mathrm{i}} \sim N(x', \sigma^2_{\mathrm{var}})$, where σ^2_{var} describes the variability in individual orientations in a given experimental condition. However, to equate the distribution of average orientations across experimental conditions, we applied a ‘correction’ to the eight samples by defining $\mathbf{z} = \mathbf{x} + \mathbf{A} * \mathbf{u}$, where $\mathbf{A}$ is an eight-by-eight matrix defined by $\mathbf{A}_{\mathrm{i,j}} = \{7/8 \; if \; i = j, -1/8 \; otherwise\}$ and $\mathbf{x}$ is a an eight-element vector where each element is x' (lower case bold: vector; upper case bold: matrix). In short, the correction ensures that the variability of the array does not change the pre-specified average orientation of the trial. Thus, $\mathbf{z}$ contains the eight orientations which were displayed on the screen and which determined response accuracy. We note that,

$$\mathbf{z}|x' \sim N(\mathbf{x}, \sigma^2_{\mathrm{var}}\mathbf{A} * \mathbf{A}') = N(\mathbf{x}, \sigma^2_{\mathrm{var}}\mathbf{A}) \tag{3}$$

because $\mathbf{A} * \mathbf{A}' = \mathbf{A}$.

The agent perceives neither x' nor $\mathbf{z}$ but eight noisy sensory samples, $\mathbf{y}$, each drawn from a Gaussian distribution centred on the true orientation of the corresponding grating, with variance, σ^2_{cont}:

$$y_i \sim N(z_i, \sigma^2_{\mathrm{cont}}) \tag{4}$$

where y_{i} is the perception of the ith grating, z_{i}, and σ^2_{cont} is the variance of the encoding noise for the current contrast condition. We assumed that the y_{i}’s are independent conditional on **z.** From the agent’s perspective, given x' and a condition specified by σ^2_{var} and σ^2_{cont}, sensory evidence $\mathbf{y}$ is distributed as:

$$\mathbf{y}|\mathrm{cond}, x' \sim N(\mathbf{x}, \sigma^2_{\mathrm{var}}\mathbf{A} + \sigma^2_{\mathrm{cont}} * \mathbf{I}) \tag{5}$$

where **I** is the identity matrix (this equation is obtained by marginalizing over **z**).

The agent bases its inference on **y** alone. However, because it knows the structure of the task, it can marginalize over the condition and x'. To make a choice, the agent calculates the probability of each category (CW or CCW) conditional on its observation **y** as follows:

$$\mathrm{p}(\mathrm{cat}|\mathbf{y}) = \frac{\mathrm{p}(\mathbf{y}|\mathrm{cat})\mathrm{p}(\mathrm{cat})}{\mathrm{norm}} = \mathrm{p}(\mathrm{cat}) \sum_{\mathrm{i}=1}^{\mathrm{n}} \frac{\int_{-\mathrm{inf}}^{\mathrm{inf}} \mathrm{p}(\mathbf{y}, x', \mathrm{cond_i}|\mathrm{cat}) dx'}{\mathrm{norm}}$$
$$= \mathrm{p}(\mathrm{cat}) \sum_{\mathrm{i}=1}^{\mathrm{n}} \frac{\int_{-\mathrm{inf}}^{\mathrm{inf}} \left(\mathrm{p}(\mathbf{y}|\mathrm{cat}, x', \mathrm{cond_i}) \cdot \mathrm{p}(x', \mathrm{cond_i}|\mathrm{cat})\right) dx'}{\mathrm{norm}}$$
$$= \mathrm{p}(\mathrm{cat}) \sum_{\mathrm{i}=1}^{\mathrm{n}} \mathrm{p}(\mathrm{cond_i}) \frac{\int_{-\mathrm{inf}}^{\mathrm{inf}} \left(\mathrm{p}(\mathbf{y}|x', \mathrm{cond_i}) \cdot \mathrm{p}(x'|\mathrm{cat})\right) dx'}{\mathrm{norm}} \tag{6}$$

where i sums over conditions (i.e. variability and contrast combinations), with n indicating the number of conditions, and $\mathrm{norm} = \mathrm{p}(\mathbf{y})$ is the normalization constant. Note that we here only consider neutral trials where the two stimulus categories have equal prior probability.

Because the sign of x' (and thereby the sign of the sum of **z**) defined the category and all conditions were equally probable, we obtain:

$$\mathrm{p}(\mathrm{cat}|\mathbf{y}) = \left\{ \begin{array}{ll} \mathrm{p}(\mathrm{cat})\mathrm{p}(\mathrm{cond}) \sum_{\mathrm{i}=1}^{\mathrm{n}} \frac{\int_{0}^{\mathrm{inf}} \left(\mathrm{p}(\mathbf{y}|x', \mathrm{cond_i}) \cdot \mathrm{p}(x'|\mathrm{cat})\right) dx'}{\mathrm{norm}} & \mathrm{cat} = \mathrm{CW} \\ \mathrm{p}(\mathrm{cat})\mathrm{p}(\mathrm{cond}) \sum_{\mathrm{i}=1}^{\mathrm{n}} \frac{\int_{-\mathrm{inf}}^{0} \left(\mathrm{p}(\mathbf{y}|x', \mathrm{cond_i}) \cdot \mathrm{p}(x'|\mathrm{cat})\right) dx'}{\mathrm{norm}} & \mathrm{cat} = \mathrm{CCW} \end{array} \right\} \tag{7}$$

Note that the terms within the integral are given in Supplementary Equations (1), (2) and (5). The agent chooses the CW category if $\mathrm{p}(\mathrm{CW}|\mathbf{y}) > \mathrm{p}(\mathrm{CCW}|\mathbf{y})$ and the CCW category if $\mathrm{p}(\mathrm{CCW}|\mathbf{y}) > \mathrm{p}(\mathrm{CW}|\mathbf{y})$. The experimenter classifies a choice as correct if it is CW and $x' > 0$ or if it is CCW and $x' < 0$. The agent's confidence in its choice is given by:

$$\mathrm{conf} = \max\{\mathrm{p}(\mathrm{CW}|\mathbf{y}), \mathrm{p}(\mathrm{CCW}|\mathbf{y})\} \tag{8}$$

We simulated behaviour of the ensemble model under different levels of random zero-mean Gaussian noise using two parameters: one parameter, $\sigma_{\mathrm{low\ contrast}}$, specified encoding noise on low-contrast trials, whereas the second parameter, $\sigma_{\mathrm{high\ contrast}}$, specified encoding noise on high-contrast trials. We used forty linearly-spaced values over the range 0.1-20 for each parameter, yielding a total of 1,600 parameter combinations. We used three fixed values of $\sigma_{\mathrm{var}}{}^2 = \{0, 4, 10\}$ that defined the variability conditions in the same way as in Experiments 1-3. We generated 40,000 simulated trials (i.e. eight gratings) for each experimental condition using Supplementary Equation (3). For each parameter combination, to generate sensory evidence **y**, we added a random sample of Gaussian noise (from a distribution whose variance is determined by the contrast level of the current condition) to each grating, as in Supplementary Equation (4). We then used Supplementary Equation (6) to compute the model's posterior belief about the categories. We computed model choices as the category with the higher posterior, and model confidence as in Supplementary Equation (7). Note that, while sensory evidence **y** depends only on one of the noise parameters

($\sigma_{\text{low contrast}}$ or $\sigma_{\text{high contrast}}$), the inference process depends on both noise parameters and on the distribution of $\mathbf{y}$ across all experimental conditions.

We thus obtained estimates of average choice accuracy and mean confidence under different experimental conditions and parameter settings by averaging choices and confidences for each experimental condition (Supplementary Fig. 2). As expected, accuracy in the low-contrast, zero-variability condition and in the low-contrast, high-variability condition depended only on the noise parameter for low contrast (panels a and b, respectively). Conversely, accuracy in the high-contrast, zero-variability condition and in the high-contrast, high-variability condition depended only on the noise parameter for high-contrast (panels e and f, respectively). Critically, the ensemble model, which does not have integration noise, shows no difference in performance across conditions with different variability but matched contrast (panel c shows difference between panels a and b, whereas panel g shows difference between panels e and f). This prediction stands in stark contrast to the pattern of accuracy observed in our data – which was explained by the addition of a noise term (integration noise) that scales with stimulus variability. Furthermore, when assessing the predicted mean confidence of the ensemble model, we find another striking difference between the model and the human data. In particular, the ensemble model is overconfident on low-contrast, zero-variability trials and underconfident on high-contrast, high-variability trials (panels d and h, respectively). In contrast, participants were overconfident on high-contrast, high-variability trials – a pattern which was captured by the noise blind model.

In sum, these simulations demonstrate that neither integration noise nor noise blindness naturally fall out of a model that makes inferences at the level of individual items.

Supplementary Figure 2

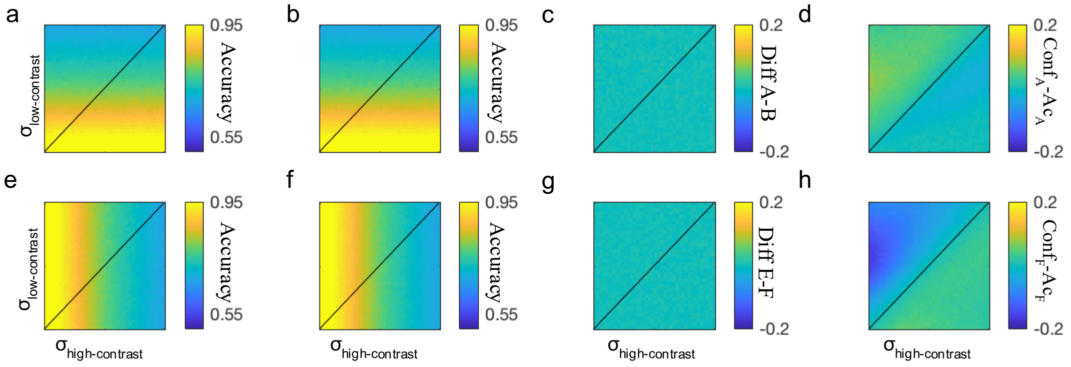

Choice accuracy and confidence calibration under an ensemble model which operates with the individual items of a stimulus array. We test in simulation choice accuracy (proportion correct) and confidence calibration (mean confidence minus proportion correct) under the ensemble model. We focus on four critical conditions of our experimental design and independently vary the amplitude of noise (σ) for stimuli of high contrast (x-axis) and low contrast (y-axis). (**a**) Accuracy in the low-contrast, zero-variability condition. (**b**) Accuracy in the low-contrast, high-variability condition. (**c**) Difference in accuracy between the low-contrast, zero-variability condition and the low-contrast, high-variability condition. (**d**) Confidence calibration in low-contrast, zero-variability condition. (**e**) Accuracy in the high-contrast, zero-variability condition. (**f**) Accuracy in the high-contrast, high-variability condition. (**g**) Difference in accuracy between the high-contrast, zero-variability condition and the high-contrast, high-variability condition. (**h**) Confidence calibration in the high-contrast, high-variability condition.

Supplementary Note 3

Overconfidence in choices

A diagnostic difference between the variability-mixer and the noise-blind models is the predicted pattern of overconfidence (i.e. mean confidence minus mean accuracy) across the key conditions of our factorial design. The variability-mixer model predicts a hard-easy effect, with overconfidence for the high-variability condition and underconfidence for the baseline condition. By comparison, while the noise-blind model also predicts overconfidence for the high-variability condition, the model predicts good calibration for the baseline condition. Indeed, as expected under the noise-blind model, participants were overconfident in the high-variability condition but well-calibrated in the baseline condition (Supplementary Fig. 3).

Supplementary Figure 3

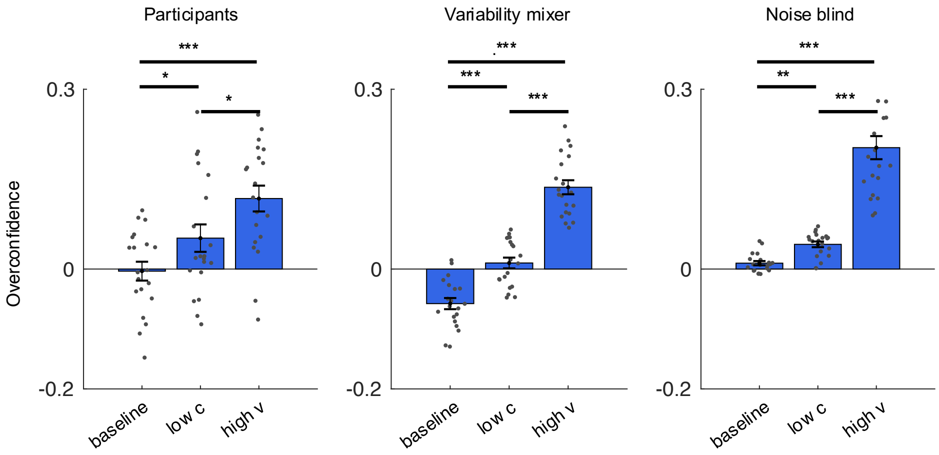

Overconfidence in choices. Participants (left) are well-calibrated in the baseline condition but overconfident in other conditions. The variability-mixer model (middle) shows underconfidence in the baseline condition but overconfidence in the high-variability condition. The noise-blind model shows good calibration in the baseline condition and overconfidence in the high-variability condition. Near-zero values indicate good calibration and non-zero values indicate bad calibration (negative: underconfidence; positive: overconfidence). Data is from neutral trials of Experiment 2 and represented as group mean ± SEM.

Supplementary Note 4

Fitting common threshold drift diffusion model to data

To test the sequential sampling account of suboptimal behaviour proposed by Zylberberg and colleagues[4], we fitted a Drift Diffusion Model (DDM) to the data from Experiments 1-2 (neutral trials). The DDM models two-choice decision-making as a process of accumulating noisy evidence over time with a certain speed, or drift-rate, until one of two choice thresholds is crossed and the associated response is executed. We assumed that the choice thresholds were fixed across experimental conditions as in the study by Zylberberg and colleagues[4]. In addition, we assumed that lower contrast led to a lower drift-rate and that higher variability led to higher noise (within-trial drift-rate variability). We implemented these mechanisms using three parameters. The first parameter depends on the contrast level and scales the drift-rate. The second parameter specifies the baseline variance of the drift-rate. Finally, the third parameter depends on the variability level

and scales the baseline variance of the drift-rate. The baseline drift-rate was proportional to the absolute difference between the average orientation and horizontal.

To find the best parameters for each participant, we minimized the sum of squared errors between empirical and predicted choice accuracy across experimental conditions. We used a genetic algorithm with a population size of 1000 individuals and a maximum generation time of 1000 generations. Comparison between empirical data and model predictions are shown in Supplementary Fig. 4. In short, the model can predict the observed choice accuracy for the different conditions (panel a), but it predicts a pattern of response times with respect to stimulus variability opposite to what we observed (panel b). As a sanity check, we show that higher evidence strength (i.e. absolute deviation of the average orientation from the category boundary) indeed increases choice accuracy and fastens response times (panels c-f).

Supplementary Figure 4

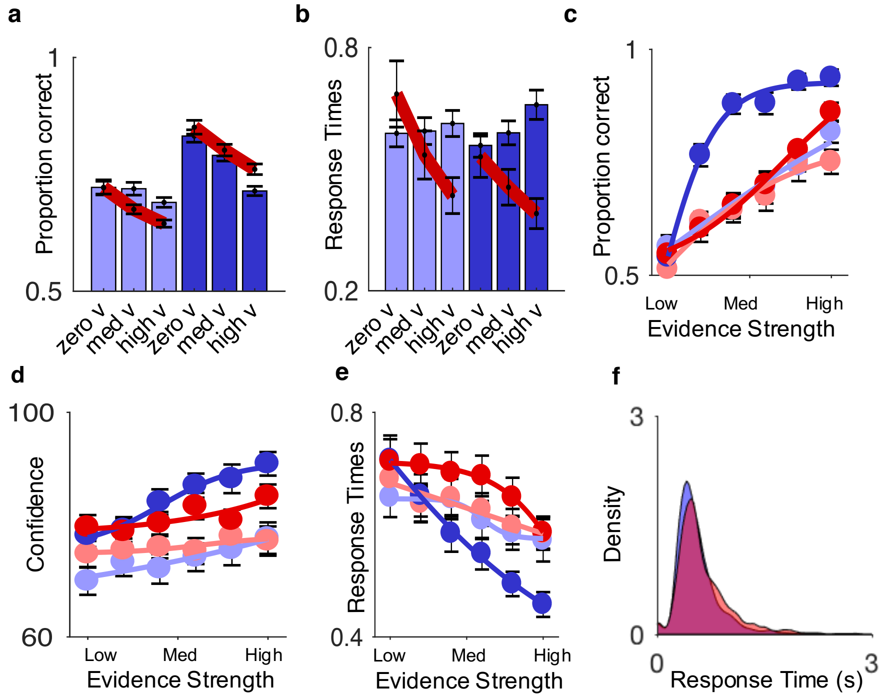

Common choice threshold in a sequential sampling model cannot explain our data. (**a**) Choice accuracy for participants (blue bars) and DDMs (red lines) is lower when contrast is low (compare pale blue and dark blue bars) and when variability is high (negative slopes as condition changes from zero-v to high-v). (**b**) Response times for participants and DDMs show opposite effects for increases in variability (positive slopes for participants but negative ones for DDMs). (**c**) Participants' choice accuracy for different levels of evidence strength (blue: low variability, red: high variability; faint colours: low contrast; dark colours: high contrast). Note that the two critical conditions, high-variability and high-contrast trials (dark red curve) and low-contrast and zero-variability trials (faint blue curve), have similar slopes. (**d**) Participants' confidence for different levels of evidence strength (same colour scheme as in panel c). (**e**) Participants' response times for different levels of evidence strength (same colour scheme as in panel c). (**f**) Collapsing response times across participants for high-contrast and zero-variability trials (blue) and high-contrast and high-variability trials (red) demonstrate that high variability is associated with slower response times (i.e. red distribution has a longer tail). For panels c-e, evidence strength is divided into quantile bins of roughly one degree of width starting at zero. Data is from neutral trials of Experiments 1-2 and represented as group men ± SEM.

Supplementary Note 5

Hierarchical drift diffusion model

To further investigate how our experimental factors affected choice formation, we employed hierarchical Bayesian estimation of subjects' DDM parameters using the HDDM toolbox[5]. To remain agnostic as to how our task affected DDM parameters, we fitted non-decision time, threshold and drift-rate separately for each condition of our factorial design (six conditions) in Experiments 1-2 (neutral trials only). The HDDM toolbox applies Markov Chain Monte-Carlo sampling to approximate posterior distributions over DDM parameters. We ran one chain with ten thousand samples, with the first one thousand samples discarded as burn-in. We extracted statistics summarising group-level posterior distributions for visualisation of DDM parameters.

Under our account, the performance cost associated with high-variability stimuli is due to additional noise during the decision process (integration noise). The simulations described in the previous section ruled out a pure threshold account in which the performance cost is due to subjects employing a common threshold across conditions. However, an alternative, but related, account is that the performance cost is due to between-condition variability in threshold. The HDDM fits rule out this alternative account: we found that only drift-rate varied between conditions, whereas decision boundaries and non-decision time were stable across conditions (Supplementary Fig. 5). In summary, both the DDM simulations and the HDDM fits indicate that the performance cost associated with high-variability stimuli is due to additional noise during the decision process – a phenomenon that we termed integration noise.

Supplementary Figure 5

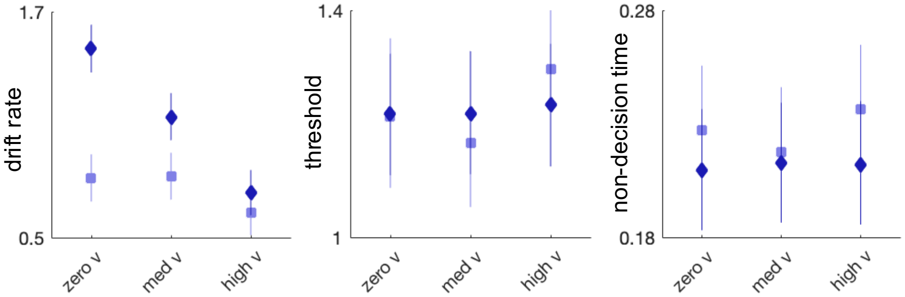

DDM parameters split by condition. Mean posterior estimates obtained from hierarchical Bayesian estimation of subjects' DDM parameters across the six conditions in Experiments 1-2 using the HDDM toolbox (light blue squares for low contrast conditions; dark blue diamonds for high contrast conditions; the variability condition is marked on the x-axis). We fitted drift-rate, threshold and non-decision time separately for each condition of our factorial design. Error bars indicate 95% confidence intervals as estimated from the posterior distributions over parameters.

Supplementary Note 6

Subsampling

We observed a decrease in performance for trials with high stimulus variability across all experiments. We attributed this decrease to integration noise – an increased difficulty for integrating variable or disparate pieces of information – which can explain both decreased choice accuracy and increased response times for high-variability stimuli. An alternative explanation of the decrease in accuracy observed on trials with high stimulus variability is that participants based

their judgments on a subset of the items in a stimulus array. Under this subsampling account, the decrease in accuracy is due to a larger mismatch between the actual average orientation of the full array and the average orientation of the sampled subset. Here we explain why subsampling is unlikely to account for the decrease in accuracy on high-variability trials, and why subsampling cannot explain noise blindness.

First, we found no effect of set-size on accuracy in Experiment 6 (Supplementary Fig. 6a) which was designed to test the subsampling hypothesis. In this experiment, the distribution of average orientations was the same for both set-sizes. If participants sampled all items, then there should be no mismatch and thereby no difference in performance between the two set-sizes. If instead subsampling did occur, it would have a larger effect on performance on trials with eight-item arrays as compared to four-item arrays. For instance, if participants sampled four items, then there would be no difference in performance between the baseline and the high-v conditions for four-item arrays (no mismatch), but there would be a difference for eight-item arrays (half of the items would be ignored). Experimentally, we found that the decrease in performance between the baseline and the high-v conditions was consistent across set-sizes and comparable to that found in the earlier experiments (Supplementary Fig. 6a). We note that another prediction for Experiment 6 is that accuracy should be higher on eight-item than four-item trials because encoding noise could be averaged out over more items. However, the data does not support this prediction. One possibility is that there is a trade-off between the number of items that are encoded and the quality with which they are encoded[6] – a trade-off which may overshadow the expected performance boost from averaging out encoding noise.

Second, we performed simulations where we varied the number of items sampled by an agent without integration noise (Supplementary Fig. 6b). Under this model, a decrease in accuracy between the baseline and high-v conditions for an eight-item array would be entirely driven by subsampling. The simulations showed that, while sampling four-items could in principle explain the decrease in accuracy between the baseline and high-v conditions for eight-item arrays, the same number of sampled items would represent the complete stimulus for arrays of four-item arrays and no expected decrease in performance should be found for high-v compared to baseline trials.

Third, we fitted a subsampling model to directly quantify the number of items sampled by each participant (n = 60; Exp1-3; neutral trials only). The model had three free parameters. The first parameter controls the noise added to each item of the array in the baseline condition. The second parameter controls the extra amount of noise added to each item in trials where the contrast is low (to capture the extent of encoding noise). Finally, the third parameter controls the number of gratings, k, that were sampled from a stimulus array; k is an integer value between one (the minimum number of items that can be sampled) and eight (the total amount of items that can be sampled). We fitted the parameters by maximising the likelihood of participants' choices using a genetic algorithm with a population size of 100 individuals and a maximum generation time of 1000 generations. Note that there is no integration noise and any reduction in accuracy for high-variability stimuli would therefore have to be due to subsampling. Even then, the fitted k was eight for most participants (Supplementary Table 2).

In summary, subsampling is unlikely to explain the decrease in accuracy for high stimulus variability, and provides no explanation for the slower responses and the apparent blindness to the performance cost associated with high stimulus variability.

Supplementary Figure 6

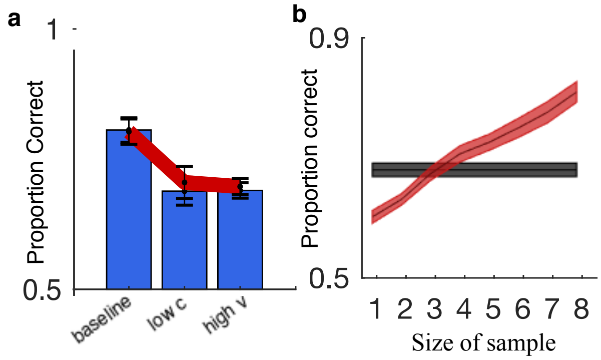

Participants are not subsampling. (**a**) Participants in Experiment 6 achieved similar levels of performance when required to integrate four items (blue bars) and eight items (red line). (**b**) Choice accuracy for participants (grey shade) and a subsampling model (yellow shade). The subsampling model, which has no integration noise and perfectly averages sampled gratings, would need to sample about four gratings before reaching the same level of choice accuracy as participants. Data is represented as group mean ± SEM.

Supplementary Table 2

Best fitting k	1	2	3	4	5	6	7	8
Number of participants	**1**	**2**	**0**	**2**	**4**	**5**	**4**	**42**

Most participants sample all eight items. Estimated number of items, *k*, sampled by participants assuming absence of integration noise.

Supplementary Note 7

Response times

In many tasks, response times provide experimenters with further information about the processes that underpin decision formation, with response times often varying with the difficulty of a decision and the confidence with which it was made.

Response times tend to be slower for harder stimuli as difficult decisions require longer deliberation. In our task, response times were indeed slower for the low-c and the high-v conditions compared to the baseline condition (t-tests; baseline<low-c: $t(39) = 2.6$, $p < .05$; baseline<high-v: $t(39) = 6.20$, $p < .001$; see Supplementary Fig. 7). However, response times were even slower for the high-v compared to the low-c condition (t-tests; low-c<high-v: $t(39) = 4.0$, $p < .001$), despite equal levels of choice accuracy in the two conditions. Analysis of the full data set confirmed that response times increased with variability (ANOVA; main effect of variability: $F(1.5,58.0) = 58.8$, $p < .001$), whereas response times did not vary directly with contrast (ANOVA; main effect of contrast: $F(1,39) = 0.1$, $p > .7$), only through an interaction with variability (ANOVA; $F(1.8,72.7) = 13.4$, $p < .001$). Overall, our argument that integration noise results from an increased difficulty for integrating variable or discordant pieces of information is supported by the slower response times observed for high stimulus variability.

There are two different ways of thinking about the relationship between confidence and response times. On one hand, we – the experimenters – can measure response times as a proxy for

participants' confidence. However, this relationship is not straightforward: quick responses might reflect rapid guesses or high certainty, and slow responses might reflect thoughtful deliberation or high uncertainty[7]. On the other hand, participants may themselves use elapsed time as a cue to how likely their decision is to be correct[8]. By recording both response times and confidence judgments, we could investigate the contribution of response times to confidence over and above correlations between response times and stimulus features (i.e. average orientation, contrast and variability). As shown by the trial-by-trial analysis of confidence presented in Fig. 3b, lower confidence was indeed associated with slower response times, suggesting that participants did utilise elapsed time as a cue to confidence. However, the analysis also shows that response times provide an incomplete description of participants' confidence as it is only one among many factors driving confidence (e.g., additional variation due to average orientation and variability).

Supplementary Figure 7

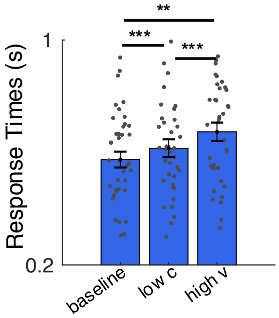

Response times for critical experimental conditions. Response times were fastest for the baseline condition, and slowest for the high-v condition. Data is from neutral trials (Exp1-2) and represented as group mean ± SEM.

Supplementary Note 8

Accuracy gain

In our task, participants had the opportunity to compensate for poor performance when the prior cue (Exp1-2) or the opt-out option (Exp3) was available. Under the noise-blindness account, the accuracy gain on such 'extra-information' trials compared to neutral trials should be higher for the low-c than the high-v condition. To test this prediction, we computed the difference in choice accuracy between these trial types: $\text{Accuracy}_{\text{gain}} = \text{Accuracy}_{\text{extra\_information}} - \text{Accuracy}_{\text{neutral}}$. Consistent with our hypothesis, accuracy gains were higher on high-c than high-v trials – a pattern which was expected under the noise-blind model but was not expected under the omniscient and the variability-mixer models (Supplementary Fig. 8).

Supplementary Figure 8

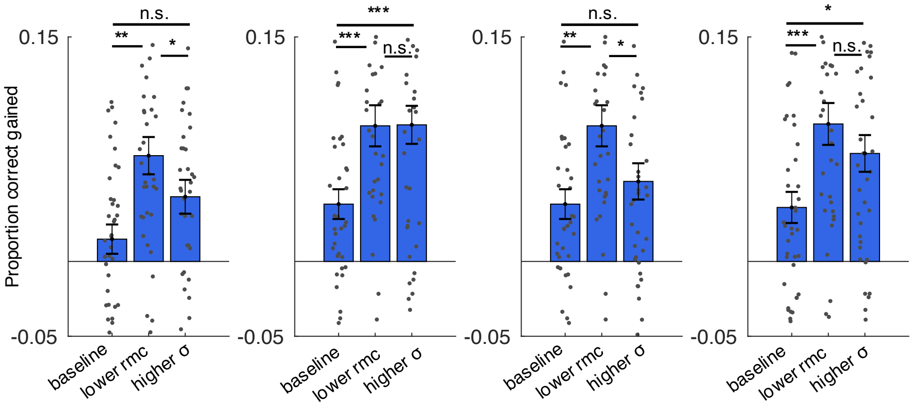

Accuracy gain for critical experimental conditions. Accuracy gain is measured as the difference in choice accuracy between 'extra-information' and neutral trials (with positive values meaning higher accuracy for 'extra-information' trials). From left to right, the panels show the accuracy gain for participants, the omniscient model, the noise-blind model, and the variability-mixer model, respectively. Data is from Exp1-3 and represented as group mean ± SEM.

Supplementary References

1. Mareschal, I. & Shapley, R. M. Effects of contrast and size on orientation discrimination. *Vision Res.* **44**, 57–67 (2004).
2. Wyart, V., Nobre, A. C. & Summerfield, C. Dissociable prior influences of signal probability and relevance on visual contrast sensitivity. *Proc. Natl. Acad. Sci.* **109**, 3593–3598 (2012).
3. Sonnemans, J. & Theo Offerman, T. Is the quadratic scoring rule behaviorally incentive compatible? (2001).
4. Zylberberg, A., Fetsch, C. R. & Shadlen, M. N. The influence of evidence volatility on choice, reaction time and confidence in a perceptual decision. *eLife* **5**, e17688 (2016).
5. Wiecki, T. V., Sofer, I. & Frank, M. J. HDDM: Hierarchical Bayesian estimation of the Drift-Diffusion Model in Python. *Front. Neuroinformatics* **7**, (2013).
6. Van den Berg, R., Shin, H., Chou, W.-C., George, R. & Ma, W. J. Variability in encoding precision accounts for visual short-term memory limitations. *Proc. Natl. Acad. Sci.* **109**, 8780–8785 (2012).

7. Pleskac, T. J. & Busemeyer, J. R. Two-stage dynamic signal detection: a theory of choice, decision time, and confidence. *Psychol. Rev.* **117**, 864 (2010).
8. Kiani, R., Corthell, L. & Shadlen, M. N. Choice certainty is informed by both evidence and decision time. *Neuron* **84**, 1329–1342 (2014).
